# Cross-kingdom inhibition of bacterial virulence and communication by probiotic yeast metabolites

**DOI:** 10.1186/s40168-021-01027-8

**Published:** 2021-03-24

**Authors:** Orit Malka, Dorin Kalson, Karin Yaniv, Reut Shafir, Manikandan Rajendran, Oshrit Ben-David, Ariel Kushmaro, Michael M. Meijler, Raz Jelinek

**Affiliations:** 1 Department of Chemistry, Ben-Gurion University of the Negev, 84105 Be’er Sheva, Israel; 2 Avram and Stella Goldstein-Goren Department of Biotechnology Engineering, Ben-Gurion University of the Negev, 84105 Be’er Sheva, Israel; 3 National Institute for Biotechnology in the Negev, Ben-Gurion University of the Negev, 84105 Be’er Sheva, Israel; 4 Ilse Katz Institute for Nanoscale Science & Technology, Ben Gurion University of the Negev, 84105 Be’er Sheva, Israel

**Keywords:** Microbiome, Quorum sensing, Vibrio cholerae, Biofilms, Probiotic microorganisms, Kluyveromyces marxianus, Tryptophol acetate

## Abstract

**Background:**

Probiotic milk-fermented microorganism mixtures (e.g., yogurt, kefir) are perceived as contributing to human health, and possibly capable of protecting against bacterial infections. Co-existence of probiotic microorganisms are likely maintained via complex biomolecular mechanisms, secreted metabolites mediating cell-cell communication, and other yet-unknown biochemical pathways. In particular, deciphering molecular mechanisms by which probiotic microorganisms inhibit proliferation of pathogenic bacteria would be highly important for understanding both the potential benefits of probiotic foods as well as maintenance of healthy gut microbiome.

**Results:**

The microbiome of a unique milk-fermented microorganism mixture was determined, revealing a predominance of the fungus *Kluyveromyces marxianus*. We further identified a new fungus-secreted metabolite—tryptophol acetate—which inhibits bacterial communication and virulence. We discovered that tryptophol acetate blocks quorum sensing (QS) of several Gram-negative bacteria, particularly *Vibrio cholerae*, a prominent gut pathogen. Notably, this is the first report of tryptophol acetate production by a yeast and role of the molecule as a signaling agent. Furthermore, mechanisms underscoring the anti-QS and anti-virulence activities of tryptophol acetate were elucidated, specifically down- or upregulation of distinct genes associated with *V. cholerae* QS and virulence pathways.

**Conclusions:**

This study illuminates a yet-unrecognized mechanism for cross-kingdom inhibition of pathogenic bacteria cell-cell communication in a probiotic microorganism mixture. A newly identified fungus-secreted molecule—tryptophol acetate—was shown to disrupt quorum sensing pathways of the human gut pathogen *V. cholerae.* Cross-kingdom interference in quorum sensing may play important roles in enabling microorganism co-existence in multi-population environments, such as probiotic foods and the gut microbiome. This discovery may account for anti-virulence properties of the human microbiome and could aid elucidating health benefits of probiotic products against bacterially associated diseases.

Video Abstract

**Supplementary Information:**

The online version contains supplementary material available at 10.1186/s40168-021-01027-8.

## Background

Probiotic milk-fermented microorganism mixtures (e.g., kefir, yogurt) are perceived and recognized as contributing to human health, and possibly capable of protecting against bacterial infections [1–4]. Co-existence and symbiosis of probiotic microorganisms are likely maintained via complex biological pathways, microorganism-secreted metabolites mediating intercellular communication, and other yet-unknown biochemical mechanisms. In particular, the means by which probiotic microorganisms inhibit proliferation of pathogenic bacteria are largely unknown; deciphering such mechanisms would be a major step towards both elucidating possible therapeutic benefits of probiotic foods as well as understanding the role of the gut microbiome in maintenance of human health and combating bacterial diseases.

Quorum sensing (QS), mediated by specific cell-secreted autoinducers, is the primary means of bacterial communication [5, 6]. QS plays major role in the synchronized production of virulence factors, such as toxins and proteases, by bacterial populations, and direct relationships between QS and pathogenesis have been demonstrated [7]. QS pathways specifically induce formation of bacterial biofilms, which confer resistance to antimicrobial molecules and drugs. Furthermore, population-wide coordination via QS is essential for some bacteria both to defend themselves and to effectively attack their hosts [8]. Interestingly, inter-species bacterially secreted QS compounds have been also identified [9, 10]. Significant efforts have been directed in recent years towards development of anti-bacterial therapeutic strategies based upon identification of antagonists or agonists in QS cascades [11, 12]. Such strategies have attracted considerable interest, as they circumvent the emergence of bacterial resistance to antibiotic compounds, a major and growing challenge in anti-bacterial therapeutics [13].

A recent study has shown that molecules secreted by a probiotic *Bacillus* strain interfered with cell-cell communication of a pathogenic bacterial species, *Staphylococcus aureus* [14]. In particular, that work pointed to QS pathways of pathogenic bacteria as possible targets for the secreted molecules. Notably, there has been no report of cross-kingdom effects upon QS pathways in probiotic microorganism populations. In this study, we identified, for the first time, a compound secreted by probiotic yeast that blocks bacterial communication and inhibit virulence of pathogenic bacteria. Specifically, we show that tryptophol acetate, secreted by *Kluyveromyces marxianus*, modulates QS of several Gram-negative bacterial pathogens. Although tryptophol acetate has been previously found in plants and algae [15, 16], this is the first report of its production by a yeast and particularly its role as a signaling molecule. The intriguing cross-kingdom communication interference we identified may be a fundamental tenet of microorganism co-existence in complex multi-population environments (such as probiotic foods and the human gut microbiome) and may contribute to development of new therapeutic strategies.

## Results and discussion

### Kefir composition

**Fig. 1:**
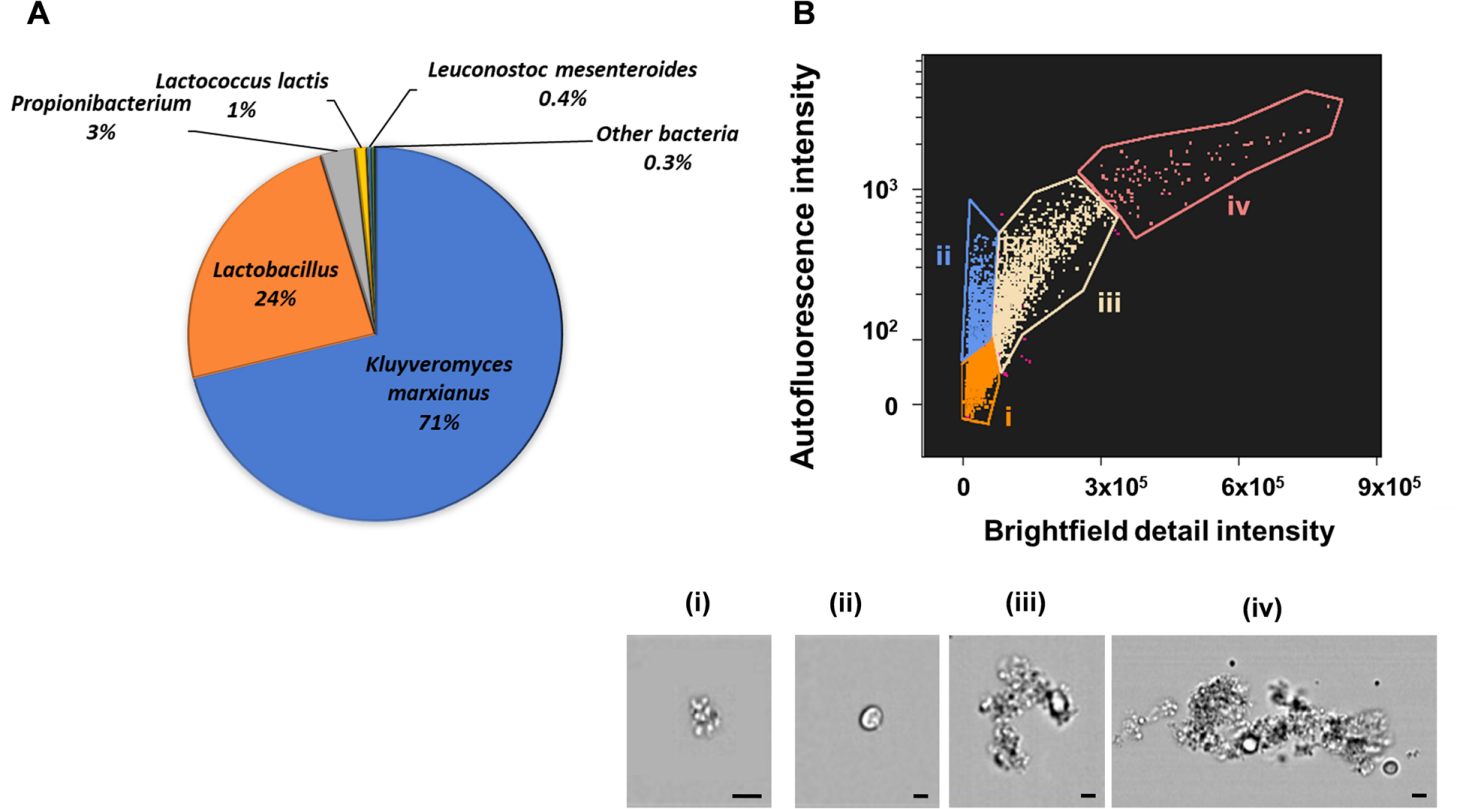
Microorganism composition of the kefir. **a** Distribution pie chart of microorganisms in the kefir, obtained through total DNA shotgun sequencing based on a BLAST comparison in the One Codex data platform for applied microbial genomics. **b** Imagestream® flow cytometry analysis depicting microorganisms size/shape distribution. The dot plot of autofluorescence vs. bright field detail intensities of 19380 events analyzed. The brightfield cell images were used to draw an initial dot plot to identify cells of interest and exclude debris. The populations corresponding to initial hand-picked galleries of images are automatically gated on dot plot and each gate is indicated with a different color. Representative microscopic images of the cell subpopulations presented in the scatterplot: (i) bacterial cells; (ii) fungal cells; (iii–iv) fungal/bacterial cell aggregates. Scale bars correspond to 5 μm

In this work, we focused on milk-fermented Tibetan kefir as the source of secreted molecules interfering with bacterial communication. The microorganism populations of the kefir, outlined according to taxonomy annotation, were determined by total DNA shotgun sequencing and are depicted in Fig. 1a. The dominant genetic constituent of the kefir—approximately 70% of total reads—was *Kluyveromyces marxianus*, 24% were *Lactobacillus* species, and the remainder comprised of other genera, including *Propionibacterium, Lactococcus lactis,* and *Leuconostoc mesenteroides* (Fig. 1a; detailed microorganism distribution is presented in Table S1). Imagestream® flow cytometry analysis showing a scatterplot of autofluorescence intensity vs. bright field detail intensity of 19,380 events, is presented in Fig. 1b. The flow cytometry data indicate that subpopulations defined as bacterial cells comprised 87% of the kefir mixture (representative microscopy image of the bacterial cells is depicted in Fig. 1b (i)), fungal cells were approximately 5% (Fig. 1b (ii)), and the remainder subpopulations of fungi/bacteria aggregates (Fig. 1b (iii–iv)). Detailed event counts are presented in Table S2.

### Effect of the kefir on bacterial quorum sensing

**Fig. 2:**
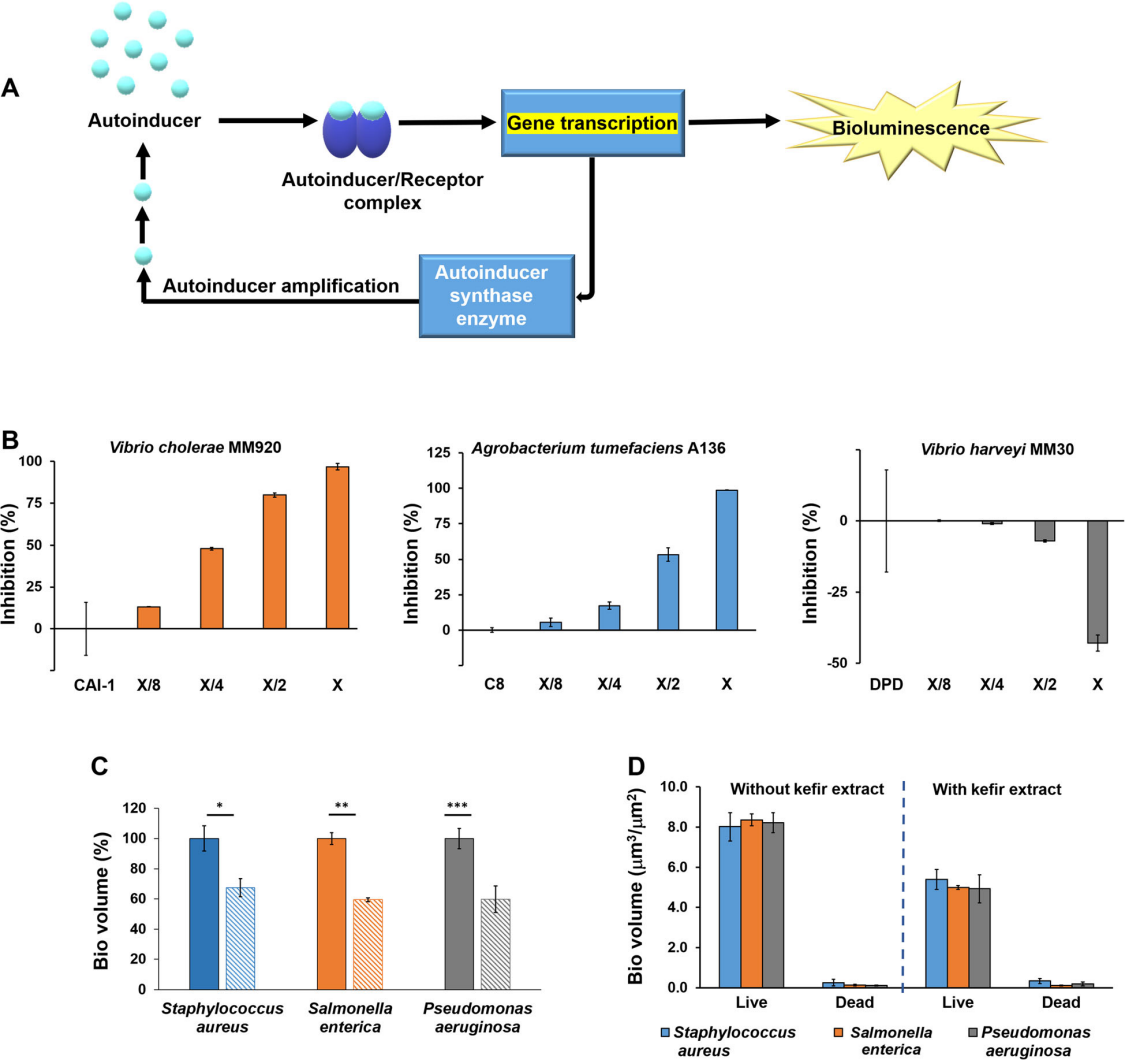
Effects of the kefir on bacterial quorum sensing and bacterial biofilms. **a** Schematic description of the generic bioluminescence induction mechanism in the reporter QS Gram-negative bacterial strains. **b** QS inhibition in the bioluminescent reporter strains *Vibrio cholerae MM920*, *Agrobacterium tumefaciens A136*; QS activation observed in the case of *Vibrio harveyi MM30*. Bioluminescence recorded upon addition of the kefir crude extract in different dilutions. The respective autoinducers (CAI-1, C8-HSL, DPD) correspond to the control experiments (no inhibition/activation of bioluminescence). **c** Bar diagrams showing biofilm volumes percentage generated in three wild-type bacterial strains upon addition of the kefir crude extract to the growth media (striped bars) and non-treated bacteria (solid bars). The graph displays the means (±SD; *n* = 3) of biofilm volume per area, generated from three independent sets of confocal fluorescence microscopy experiments calculated through the IMARIS software. *p* value: * *p* < 0.12, **p < 0.01, ***p < 0.002 calculated by ANOVA followed by Tukey’s post hoc analysis. **d** Viability analysis of bacterial cells in the biofilms. *Staphylococcus aureus*, *Salmonella enterica,* and *Pseudomonas aeruginosa* viable cells were stained in green while dead cells were stained red with the BacLight® Dead/Live Kit

Bioluminescence assays employing reporter strains lacking the gene encoding the enzyme for QS autoinducer synthesis (CAI-1) [17] were performed to determine the effect of the kefir on bacterial QS (Fig. 2). A scheme depicting the principle of the bioluminescence QS analysis is illustrated in Fig. 2a. Essentially, high concentrations of autoinducer molecules bind to specific receptors which consequently activate the intracellular transcription machinery which stimulates expression of bioluminescence genes (i.e., high bioluminescence emission indicates quorum sensing activation in the system).

In the experiments outlined in Fig. 2b, bioluminescent QS reporter strains and the QS autoinducers for each bacterial species [18–20] were incubated in the presence of crude kefir extract, thereby allowing quantitative assessment of QS inhibition or activation. Importantly, Fig. 2b demonstrates that the kefir crude extract interfered with QS pathways of all three bacterial species examined. In case of the *Vibrio cholerae* MM920 mutant lacking the ability to synthesize its CAI-1 autoinducer, the kefir extract had a significant quorum sensing inhibitory (QSI) effect, as illustrated by the direct correlation between kefir crude extract dilution and attenuation of bioluminescence [the baseline in the graphs in Fig. 2b corresponds to the luminescence recorded upon addition of the autoinducers alone (i.e., no inhibition of QS) [17, 21].

The kefir extract similarly had a concentration-dependent QSI effect in case of *Agrobacterium tumefaciens* A136, in which the QS pathway was induced by its 3-oxo-octanyl homoserine lactone autoinducer [20] (Fig. 2b). Interestingly, the results of the *Vibrio harveyi* MM30 bioluminescence assay utilizing the (4*S*)-4,5-dihydroxy-2,3-pentanedione (*S*-DPD) autoinducer [22] appear to show that the kefir extract induced quorum sensing activation (QSA) in all dilutions (Fig. 2b). In addition, tryptophol acetate did not interfere in the extent of proliferation of *V. cholerae* bacterial cells, nor other bacterial strains tested in this work (e.g., Fig. 2), including *V. harveyi*, *A. tumefaciens*, and *P. aeruginosa* (Figures S1, a–c and S2 ). As the bioluminescence assays in Fig. 2b indicated that substances in the kefir extract affect QS pathways (inhibition or activation) of different bacteria, we further investigated whether the kefir extract could influence formation of biofilm matrixes assembled by pathogenic bacteria (Fig. 2c). Biofilms are rigid proteinaceous/oligosaccharide matrixes which function as protective layers and virulence factors of diverse bacteria [23].

Importantly, QS cascades are fundamental processes in biofilm formation [24]. Indeed, our results reveal significant inhibitory effect of the kefir extract upon biofilms assembled by the prominent pathogenic bacteria *Pseudomonas aeruginosa*, *Salmonella enterica*, and *Staphylococcus aureus* (Fig. 2c). Notably, the quantitative analyses demonstrate reductions of between 30 and 40% in biofilm volumes induced by co-incubation of the bacteria with the kefir extract, as compared to untreated bacteria. Cell-viability assays confirmed that the kefir extract did not adversely affect bacterial cell proliferation and viability, thus indicating that disruption of cell-cell communication is the likely factor contributing to kefir-induced biofilm inhibition (Fig. 2d). It should be noted, however, that other constituents in the kefir extract may as well play a role in biofilm inhibition.

### Kefir-extracted molecule interferes with *Vibrio cholerae* communication pathways

**Fig. 3:**
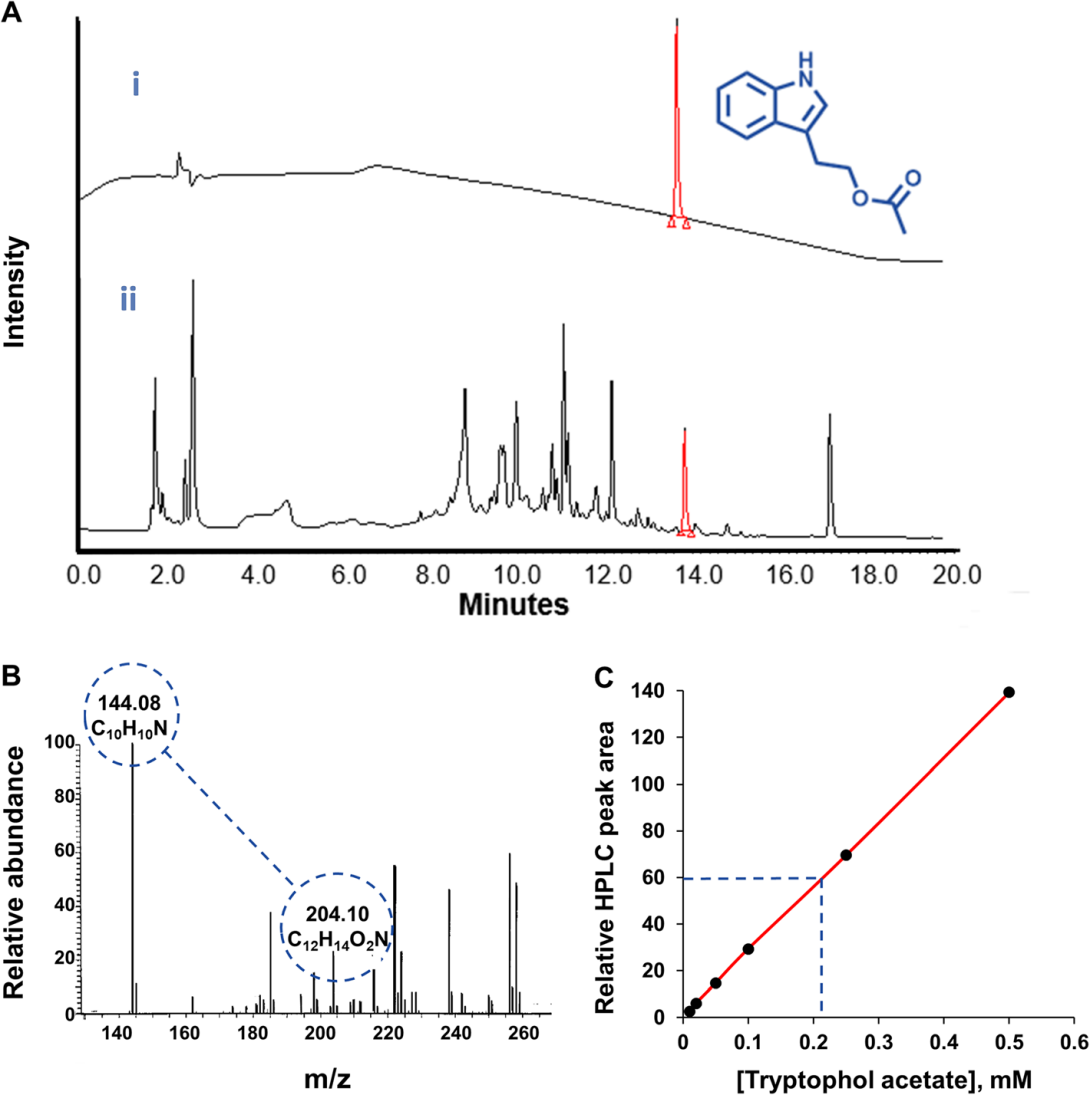
Identification of tryptophol acetate secreted by *Kluyveromyces marxianus.* **a** HPLC chromatogram of (i) tryptophol acetate extracted from the *K. marxianus* monoculture crude with a retention time of 13.84 min (peak indicated in red); tryptophol acetate molecular structure is indicated. (ii) Tryptophol acetate identified in the kefir crude extract (peak shown in red at the same retention time). **b** MS spectrum acquired in positive enhanced mass spectrometry for the identified tryptophol acetate peak in the kefir crude extract (between 13.72 and 14.05 min). **c** Calibration curve of tryptopol acetate in the kefir crude extract constructed with the synthetic compound. The broken lines indicate the concentration of tryptophol acetate in the kefir extract (210 μM)

**Fig. 4:**
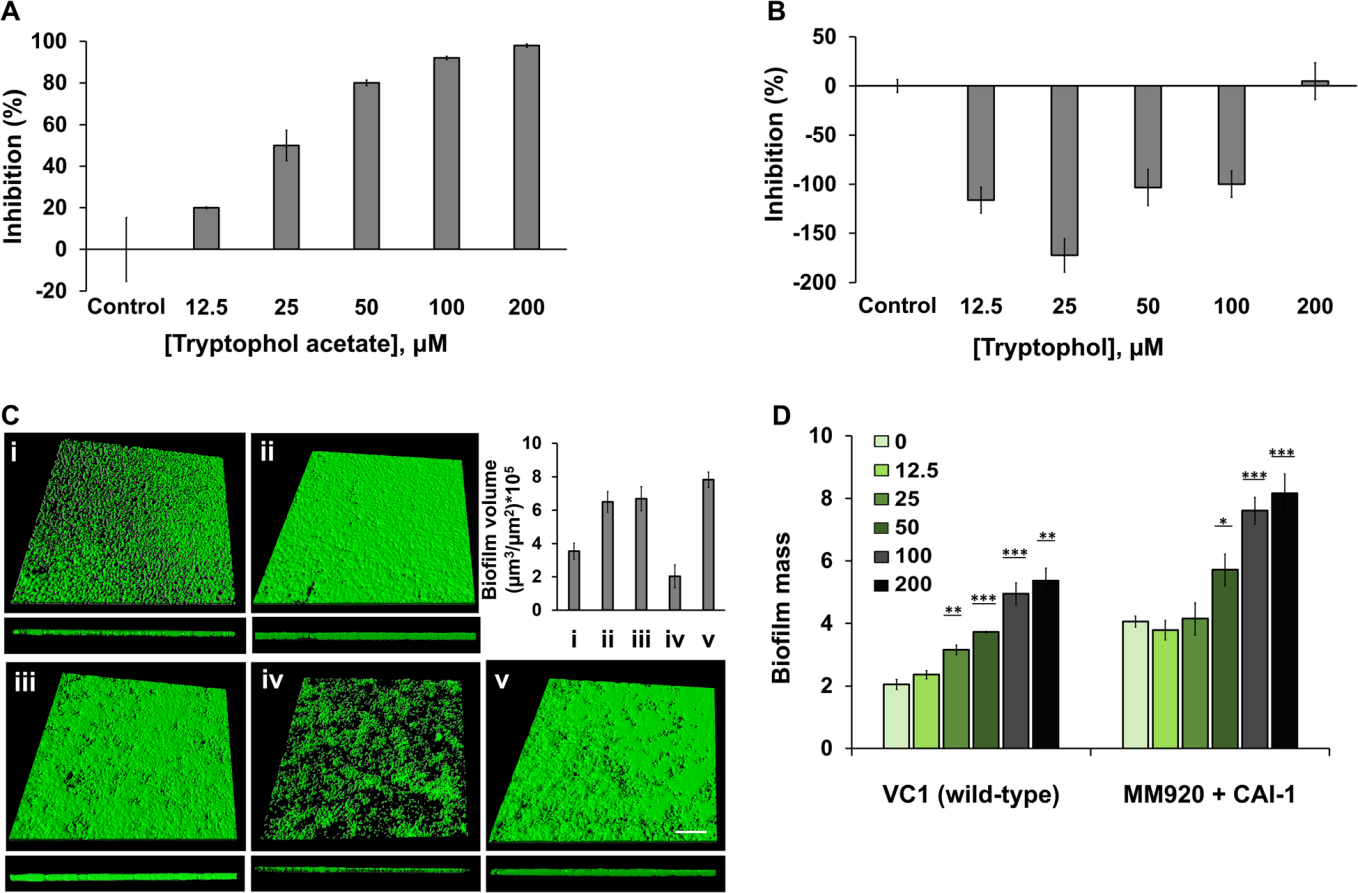
Effects of tryptophol acetate on *Vibrio cholerae* quorum sensing and biofilm assembly. **a** Tryptophol acetate concentration-dependent inhibition of the CAI-1 QS system in *V. cholerae* MM920. Experiments were performed in triplicate and error bars represent standard deviation of the mean. **b** Concentration-dependent effect of tryptophol on the CAI-1 QS system in *V. cholerae* MM920. Experiments were performed in triplicate and error bars represent standard deviation of the mean. **c** Confocal fluorescence microscopy z-stacks showing *V. cholerae* biofilms. Excitations were at 488 nm and 561 nm; emission 490–588 nm and 604–735 nm, respectively. *V. cholerae* viable cells were stained in green while dead cells were stained red with the BacLight® Dead/Live Kit. Sizes of the biofilm images are 500 μm × 500 μm. (i) *V. cholerae* VC1 (wild-type); (ii) *V. cholerae* VC1 grown in the presence of 100 μM tryptophol acetate; (iii) *V. cholerae* MM920 mutant*;* (iv) *V. cholerae MM920* incubated with 900 nM CAI-1; (v) *V. cholerae* MM920 incubated with both 900 nM CAI-1 and 100 μM tryptophol acetate. The graph (top right) displays the means (±SD) of biofilm volume per area, generated from three independent sets of confocal fluorescence microscopy experiments calculated through the IMARIS software. **d** *V. cholerae* biofilm mass analysis at different concentrations of tryptophol acetate (μM) obtained through crystal violet staining (The concentrations in μM are indicated by the different bar colors). Biofilms were stained after 24-hr growth. Error bars indicate the standard deviations of 4 measurements. *p < 0.001, **p < 0.0001, ***p < 0.000001 versus the control calculated by ANOVA followed by Tukey’s post hoc analysis

**Fig. 5:**
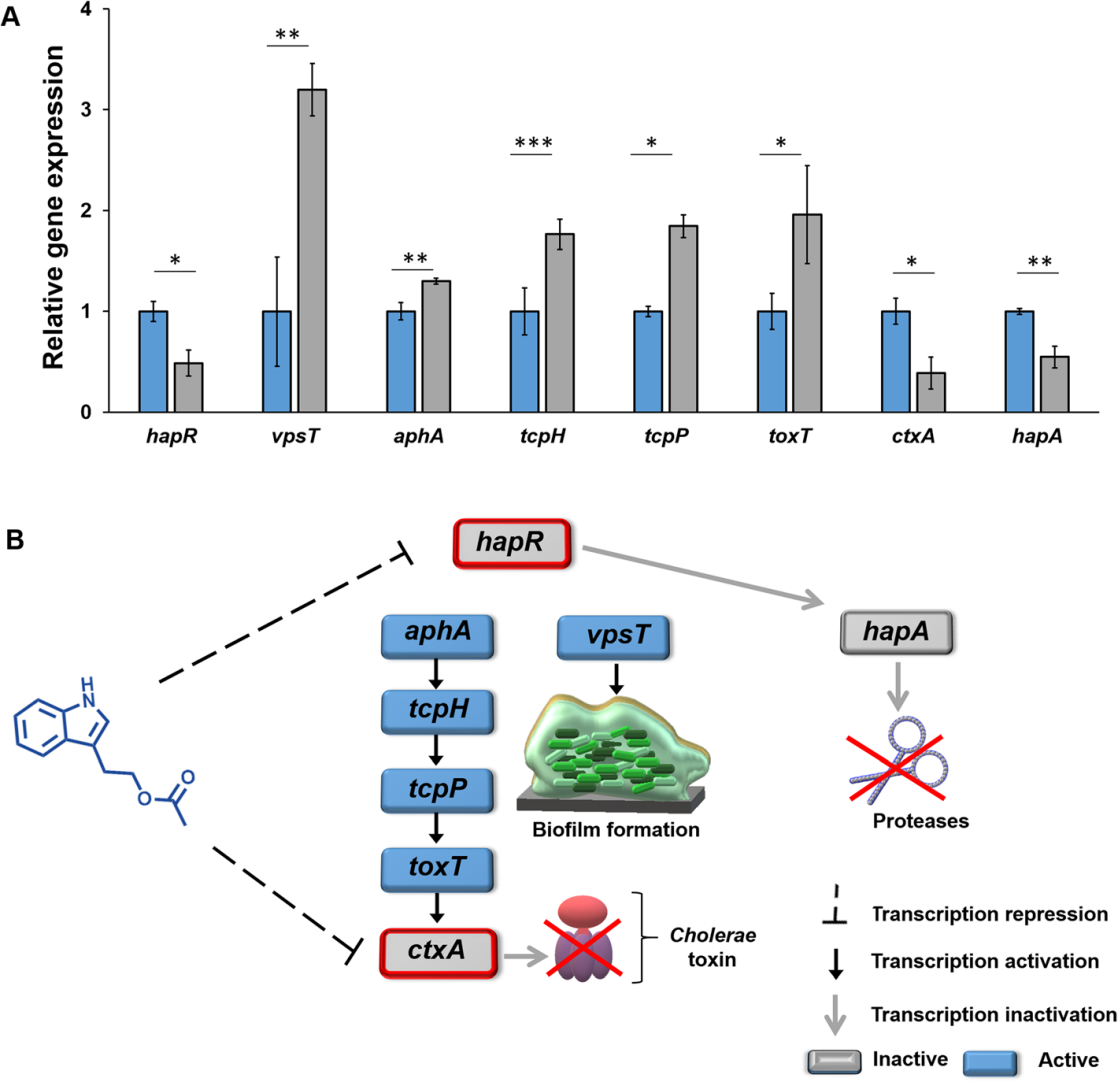
Effect of tryptophol acetate on the expression of genes associated with quorum sensing in *V. cholerae*. **a** Relative gene expression in *V. cholerae* VC1 WT assessed by RT-qPCR. The blue bars correspond to bacteria untreated with tryptophol acetate, while the grey bars indicate gene expression levels recorded following addition of the compound. The relative magnitude of gene levels was defined as the copy number of cDNA of genes in the QS pathway normalized in relation to the expression of a reference housekeeping gene not affected by the treatment. Error bars indicate standard deviations of four independent cultures. *p < 0.002, **p < 0.0001, ***p < 0.05 versus the untreated bacteria calculated by ANOVA followed by Tukey’s post hoc analysis. **b** Scheme depicting the effects of tryptophol acetate upon QS gene regulation of biofilm formation and virulence of *V. cholera*e in high cell density conditions (as recorded in the RT-qPCR experiments). The solid black arrows indicate transcription activation, the grey arrows indicate inactivation, while the dashed lines account for transcription repression induced by tryptophol acetate. Genes shown in grey background with red borders—*hapR* and *ctxA*—were downregulated by tryptophol acetate, while blue backgrounds account for genes that were upregulated due to repression of *hapR* (mimicking low cell density conditions), see text

To characterize the molecular constituents in the kefir that contribute to QS interference and disruption, we applied comprehensive screening using column chromatography. The chromatography experiments allowed identification of a small molecule secreted by *K. marxianus*—tryptophol acetate (Fig. 3 and Figure S3)—which exhibited remarkable anti-QS activities (Figs. 4 and 5, below). Notably, our focus was on *K. marxianus* metabolites since this fungus was the predominant microorganism constituent in the kefir. High performance liquid chromatography (HPLC) results show the peak ascribed to tryptophol acetate extracted from pure *K. marxianus* culture (Fig. 3a (i)). A corresponding peak having the same retention time is clearly apparent in the chromatogram of the whole kefir crude extract (Fig. 3a (ii)), indicating that tryptophol acetate was also secreted in the microorganism mixture comprising the kefir.

The molecular weight of the extracted tryptophol acetate was determined by mass spectrometry (MS) in positive ion mode [25], showing m/z peaks of 144.08 and 204.10 Da (Fig. 3b). The peak in m/z 144.08 is due to dissociation of the 204.10 Da molecule, showing a characteristic pattern in the mass spectrum (according to NIST Mass Spectrometry library identification data, ID: 144123). The isolated preparative HPLC fraction was further characterized by gas chromatography–mass spectrometry (GC-MS) and by ^1^H and ^13^C spectroscopy (Figure S3), confirming the molecular structure. We further determined that the concentration of tryptophol acetate in the kefir biomass was approximately 210 μM, based on a calibration curve employing a synthetic tryptophol acetate standard (Fig. 3c). Tryptophol acetate is likely produced through metabolic pathways as derivatives of tryptophol, fungal metabolite participating in fungal cell-cell communication [26].

Following identification of the compound secreted by *K. marxianus* in the kefir, we investigated the specific anti-QS activities of tryptophol acetate in the case of *Vibrio cholerae*, a major waterborne human gut pathogen [27] (Fig. 4). Tryptophol acetate was synthesized via conventional procedures and its purity (99.9%) was verified by MS and NMR. The effect of tryptophol acetate on the quorum sensing reporter strain *V. cholerae* MM920, the luminescent *V. cholerae* strain lacking the gene encoding the enzyme for the QS autoinducer CAI-1 [17], was determined (Fig. 4a). This strain has been widely employed for studying QS regulation, particularly compounds blocking CqsA, a prominent CAI-1 autoinducer synthase [28]. Indeed, Fig. 4a reveals dramatic concentration-dependent inhibition of *V. cholerae* QS by tryptophol acetate; almost complete blocking of the CAI-1 QS cascade was apparent upon incubation of the bacteria with 200 μM of the compound (the calculated IC50 was 22.8 ±3.7 μM). Moreover, addition of 200 μM tryptophol acetate to *V. cholerae* MM920 cultured medium had no appreciable effect on bacterial growth (Figure S1, d). We additionally tested the effect of tryptophol (previously shown to partake in fungal cell communication [26]) upon *V. cholerae* MM920 at a similar concentration range as the tryptophol acetate (Fig. 4b). Importantly, the bar diagram in Fig. 4b reveals that, different than tryptophol acetate, tryptophol did not inhibit *V. cholerae* QS, but rather enhanced QS in concentrations lower than 200 μM. This effect is probably linked to tryptophol being a cell-cell communication molecule in yeasts [26].

The effect of tryptophol acetate on *V. cholerae* biofilms, the crucial component in their proliferation and pathogenicity, is illustrated in Fig. 4c, d. Representative confocal fluorescence microscopy images attest to the significant biofilm enhancement induced by tryptophol acetate, consistent with the anti-QS effect of the compound (e.g., Fig. 4a). In the case of *V. cholerae* VC1 wild-type (WT)*,* the biofilm matrix (without addition of tryptophol acetate) appeared thin and non-uniform (Fig. 4c (i)), ascribed to the reciprocal relationship between functioning QS pathways in the *V. cholerae* VC1 WT strain and assembly of the biofilm matrix by this bacterium [29]. Indeed, contrary to most other pathogenic bacteria, *V. cholerae* responds to the accumulation of QS autoinducers in high cell densities with the repression rather than activation of biofilm formation and virulence factors [29]. A significantly denser biofilm was formed upon incubation of the proliferating bacteria with tryptophol acetate (100 μM, Fig. 4c (ii)). This result is ascribed to inhibition of the QS CAI-1 cascade by tryptophol acetate (e.g., Fig. 4a).

A similar dramatic effect of tryptophol acetate on biofilm assembly is apparent in the case of the *V. cholerae* MM920 mutant (Fig. 4c (iii–v)). The biofilm of the mutant strain alone appeared thick and dense (Fig. 4c (iii)) due to absence of the CAI-1 QS cascade [17]. However, addition of the CAI-1 autoinducer to the bacterial growth medium reintroduced QS thereby disrupting biofilm uniformity and integrity (Fig. 4c (iv)). Notably, co-addition of tryptophol acetate and CAI-1 gave rise to a dense and uniform biofilm layer (Fig. 4c (v)), reflecting inhibition of CAI QS pathway.

The variations in biofilm volumes apparent in the fluorescent microscopy images in Fig. 4c (i–v) are illustrated quantitatively (through application of 3D visualization processed using IMARIS software) in the bar diagram in Fig. 4c, top right. Dose-response biofilm mass analysis carried out through application of the crystal violet (CV) assay [30], depicted in Fig. 4d, corroborates the fluorescence microscopy (Fig. 4c), providing additional evidence for QS inhibition by tryptophol acetate. Overall, given the fact that activation of QS in *V. cholerae* leads to reduced biofilm formation, the increased biofilm volumes and mass recorded upon addition of tryptophol acetate indicate that the molecule affects direct inhibition of QS pathways in *V. cholerae.*

### Modulation of quorum sensing genetic pathways of *V. cholerae* by tryptophol acetate

Since enhanced biofilm generation by *V. cholerae* goes together with increasing virulence of this pathogenic bacterial species (primarily secretion of the cholerae toxin, CT) [31], we investigated the effect of tryptophol acetate upon the genetic mechanisms associated with QS and virulence in *V. cholerae*. Accordingly, we carried out real time–quantitative PCR (RT–qPCR) analysis evaluating gene expression of *V. cholerae* VC1 WT in high cell density conditions (Fig. 5). Indeed, the RT-qPCR results provide evidence for specific effects of tryptophol acetate (at a concentration of 100 μM) on genes associated with QS, biofilm, and virulence regulation of *V. cholerae* (the pertinent gene cascade and the effects of tryptophol acetate on gene regulation are shown in Fig. 5b), accounting for the phenotypic changes induced by the compound (i.e., Fig. 4). Specifically, the RT-qPCR results in Fig. 5a indicate significant downregulation of *hapR* by tryptophol acetate. This result is notable, since *hapR* repression occurs at low cell density conditions [32, 33] (gene regulation pathways of *V. cholerae* in low and high cell densities, respectively, are presented in Figure S4). Thus, mimicking low cell density through *hapR* downregulation may account for the enhanced biofilm generation [29, 34] as observed upon incubation of *V. cholerae* with tryptophol acetate (i.e., Fig. 4b, d).

Repression of *hapR* is also consistent with the reduced bioluminescence occurring upon incubating tryptophol acetate with *V. cholerae* MM920 (Figs. 2a and 4a) since the luciferase is associated with expression of this gene [35]. Lower expression of *hapR* further accounts for the significant upregulation of *vpsT* by tryptophol acetate (Fig. 5a, cascade shown in Fig. 5b). Interestingly, tryptophol acetate also reduced expression of *hapA* (Fig. 5a), a downstream gene regulated by *hapR* [36]. Downregulation of *hapA* may also point to potential therapeutic benefits of the kefir, since the HapA protein is associated with a variety of adverse symptoms induced by *V. cholerae*, such as fluid generation and diarrhea [33]. Additional regulatory effects of tryptophol acetate on other gene and protein constituents of QS pathways in *V. cholerae* may occur.

Mimicking low cell density for *V. cholerae* following addition of tryptophol acetate is expected to promote genetic cascades leading to enhanced virulence [36] (i.e., upregulation of the genes in the *aphA* pathway, Fig. 5b). Indeed, the RT-qPCR experiment in Fig. 5a demonstrates that tryptophol acetate induced upregulation of *aphA, tcpP*, *tcpH*, and *toxT* associated with *V. cholerae* toxin production. Surprisingly, however*, ctxA*, a virulence-inducing gene that is downstream in the *aphA-*induced virulence cascade [37], was in fact significantly repressed by tryptophol acetate (Fig. 5a).

**Fig. 6:**
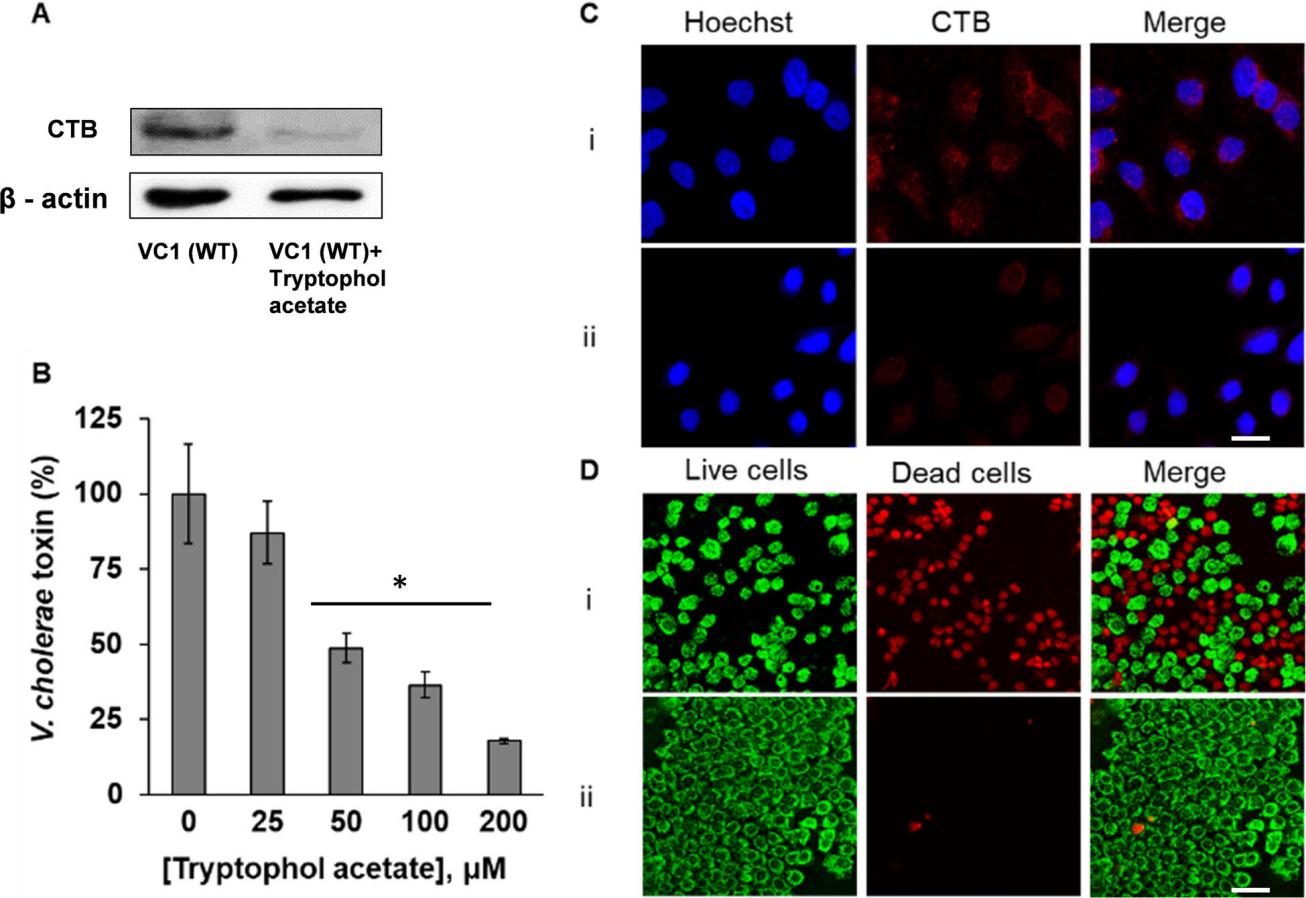
Effect of tryptophol acetate on *V. cholerae* toxin secretion. **a** Cholerae Toxin B (CTB) level expression analyzed by anti-CTB western blotting. *V. cholerae* bacterial cells (VC1 WT strain) were cultured for 16 h at 30 ^o^C without tryptophol acetate, and in the presence of 100 μM tryptophol acetate. The treated and untreated bacterial cultures were used to isolate the CTB (see “Methods” section). The samples were immunoblotted for CTB protein levels. Beta-actin used as loading control. **b** *V. cholerae* toxin production upon adding different concentrations of tryptophol acetate to the *V. cholerae* growing medium quantified with ELISA assay for CTX using GM-1 (the CTX receptor). The Y axis corresponds to percentage of CTX production in the growing medium. Error bars indicate standard deviations of four independent cultures. *p < 0.0001, versus the untreated bacteria calculated by ANOVA followed by Tukey’s post hoc analysis. **c** Confocal fluorescence microscopy images of HeLa cells grown for 16 h and exposed to extraction from VC1 WT cell lysate in the absence (i) and presence (ii) of 100 μM tryptophol acetate for 2 h. Cells were fixed and labeled with monoclonal anti-cholerae toxin, subunit B antibodies (red-excitations were at 633; emission 681 nm) and imaged. Nuclei were visualized upon co-staining of the cells with Hoechst 33342 (blue-excitations were at 405 nm; emission 445 nm). Individual channels and merged confocal images are shown. Scale bar: 50 μm. **d** Confocal fluorescence microscopy images showing the effects of CT extracted from VC1 WT cell lysate; cells were grown in the absence and presence of 100 μM tryptophol acetate. The HeLa cells were treated with CT for 16 h at 37 ^o^C and CO_2_ conditions. Propidium iodide staining (red) indicates dead cells and Syto 9 staining (green) indicates viable cells. Excitations were at 488 nm and 561 nm; emission 490–588 nm and 604–735 nm, respectively. (i) Viability staining of HeLa cells exposed to CT. (ii) Viability staining of HeLa cells exposed to CT under treatment of 100 μM tryptophol acetate. Scale bar: 100 μm

*ctxA* downregulation together with repression of *hapR* by tryptophol acetate (Fig. 5) may have significant physiological implications as lower pathogenicity and virulence of *V. cholerae*. Accordingly, we further examined the effect of tryptophol acetate upon *V. cholerae* virulence. Figure 6 examines the production of the B subunit of the cholerae toxin (CTB)—the virulence factor of *V. cholerae* regulated by *ctxA* [38]. Figure 6a shows western immunoblotting performed on VC1 WT strain cultured either in the presence or absence of 100 μM tryptophol acetate (no autoinducer added since the experiment utilized the wild-type strain). Indeed, the representative western blot in Fig. 6a demonstrates that the expression of CTB was significantly inhibited in the presence of tryptophol acetate in the medium. An ELISA assay for CTB production using GM-1 (the CTB receptor) from filtered supernatant of wild-type *V. cholerae* (Fig. 6b) similarly attests to a direct relationship between tryptophol acetate inhibition and toxin secretion by the bacteria.

The confocal fluorescence microscopy images in Fig. 6c illuminate the extent of CTB secretion by *V. cholerae* through binding of the toxin onto the surface of HeLa cells. In the experiments, the HeLa cells were incubated with CTB extracted from VC1 WT strain cell lysate, in the absence and presence of 100 μM tryptophol acetate. The cells were incubated for two hours with the toxins to allow binding to the cell-surface GM1 receptors, and subsequently stained with a fluorescently labeled monoclonal anti-CTB antibodies. The confocal fluorescence microscopy images in Fig. 6c (middle column) clearly show that cell medium obtained from *V. cholerae* bacteria treated with tryptophol acetate contained much lower concentration of CTB (i.e., significantly less staining upon incubation with CTB-antibody, Fig. 6c (ii)).

Tryptophol acetate induced inhibition of CTB secretion was further dramatically manifested by the cell viability assay in Fig. 6d. In the experiment, HeLa cells were transfected with CTX extracted from *V. cholerae* VC1 WT strain grown with or without tryptophol acetate (100 μM concentration), and subsequently co-labeled with the fluorescence markers SYTO 9 (green, staining live cells), and propidium iodide (PI, which stains dead cells in red). As apparent in the confocal fluorescence microscopy images in Fig. 6d, while significant cell death was induced by medium extracted from the control *V. cholerae* growth (abundant red staining, Fig. 6d (i)), dramatically lower cell death was recorded in HeLa cells transfected with a medium extracted from *V. cholerae* treated with tryptophol acetate (Fig. 6d (ii)). Together, the experiments depicted in Fig. 6 demonstrate that tryptophol acetate significantly reduced CTB secretion by *V. cholerae*, corroborating the genetic analysis in Fig. 5, and underscoring a remarkable virulence inhibition effect by the *K. marxianus*—secreted compound.

## Conclusions

This study reports interference with quorum sensing pathways in human pathogens induced by a probiotic yeast- *K. marxianus*. We identified a specific compound—tryptophol-acetate—secreted by *K. marxianus,* which disrupted CAI-1 QS cascades in *V. cholerae,* significantly modified *V. cholerae* biofilm formation and morphology, and reduced bacterial virulence. These phenotypic effects are ascribed to upregulation or downregulation of genes associated with the QS cascades of *V. cholerae*. These results are notable, since this is the first demonstration that QS in human pathogenic bacteria can be modulated by molecules secreted by probiotic yeast. Furthermore, tryptophol acetate has not been associated previously with bacterial QS attenuation. Our findings suggest that distinct symbioses in multi-microorganism populations may be maintained by secreted QS-modulating molecules. Such cross-kingdom QS interfering molecules may play important roles both in fermented milk products, in the digestive system of a person consuming the mixtures, and possibly also in the gut microbiome in general. These potentially universal effects may account for pathogen-combating properties of the human microbiome and could aid elucidating health benefits of probiotic microorganism products.

## Methods

### General information

The solvents Ethyl acetate (EA) and dichloromethane (DCM) were purchased from Biolab (Israel**).** Hexane, DMSO, Dulbecco’s phosphate-buffered saline (PBS X1), Trifluoroacetic acid (TFA), Petroleum ether (PE), and bovine serum albumin (BSA) were purchased from Sigma-Aldrich. Formic acid HPLC grade was purchased from Supelco. Bacterial and cells media: Lennox LB broth (L3022, Sigma-Aldrich); Miller LB broth (L3522, Sigma-Aldrich)**;** Dulbecco’s modified Eagle’s Medium (DMEM, D5796, Sigma-Aldrich).

### Kefir culturing

The kefir grains were offered originally by a private household that agreed to use them in this study. In the laboratory, 50 g of the kefir grains inoculated into a 1000 mL flask containing 800 mL of pasteurized cow milk was covered with sterile gauze. The mixture was subsequently cultivated at 28 ^o^C for 24 h. Subsequently, the kefir was filtered to separate the grains from the fermented milk. The cultured microorganisms were sent for sequence analysis. To generate biomass crude extract of the kefir, the fermented milk was centrifuged at 1100×*g* for 10 min to separate the precipitate and supernatant. Five hundred milliliters of the supernatant was then moved to a separating funnel and mixed with 500 mL EA. The organic phase was separated from the supernatant and transferred to a round bottom flask to evaporate all the solvent until a solid residue was obtained, containing the kefir organic molecules. The extraction was repeated three times.

### All metagenome sequencing of the kefir

Genomic DNA from kefir cultures was extracted using a PowerSoil® DNA Isolation Kit (MoBio Laboratories, Loker Avenue West Carlsbad, CA, USA), according to the manufacturer’s instructions. Total DNA was sent to DNA Services (DNAS) Facility, at the Research Resources Center, the University of Illinois at Chicago (UIC) for shotgun sequencing. Sequences method Nextera XT with the sequence of paired-end 2 × 150 reads illumine Nex-Keg 500 sequencer was performed. The reads were uploaded as FASTQ to One Codex website and were analyzed for metagenomics taxonomic annotation results.

### Microbial culturing

*Kluyveromyces marxianus* strain HA 63 [ATCC, NRRL Y-8281, CBS 712] was cultured in 300 mL of yeast malt broth (Y3752, Merck) and grown at 30 °C for 24 h and agitation at 100 rpm.

*Agrobacterium tumefaciens* A136 (ΔTi plasmid) containing plasmids pCF218 (overexpressing the TraR protein, which activates the *traI* fusions in response to N-acyl-homoserine lactones) and pMV26 which contains the *traI* promoter fused to the *luxCDABE* operon of *V. harveyi* [39]. Bacterial culture was grown in Luria−Bertani broth (LB; Miller’s broth) supplemented with 25 μg/mL of kanamycin and 4.5 μg/mL of tetracycline at 28−30 °C for 24 h.

*Vibrio harveyi* strain MM30 [40] and *Vibrio cholerae* strain MM920 (*ΔcqsA, ΔluxQ*, pBB1) [17] were grown in LB Broth (Lennox) supplemented with 5 mg/L tetracycline at 30 °C for 24 h.

*Vibrio cholerae* VC1 wild-type strain was grown in LB Broth (Lennox) at 30 °C for 24 h; *Staphylococcus aureus* wild-type group IV strain [41], *Salmonella enterica* ATCC 13076 [42], and *Pseudomonas aeruginosa* PA01 wild-type strain [42] were incubated for 24 h in LB broth medium at 37 °C.

All the bacterial strains and autoinducers that activate their QS systems (CAI-1; C8−HSL and DPD) were provided by Prof. Michael M. Meijler, Ben Gurion University of the Negev, Israel.

### Imaging flow cytometry

The kefir was diluted 1:10 with water and analyzed by Image Stream X Mk II (Amnis Corporation, Seattle, WA, USA). The initial identification of in-focus images of microorganisms was conducted using the system default mask of bright-field and gradient root mean square scores. The software uses algorithms based on the pixel intensity and variation in an object image frame; essentially, the algorithm employs pixel intensity to spatially distinguish the microorganism’s cells from the surrounding background [43]. A default mask of BF (Bright field) was used to identify the microorganisms in focus and plot the Gradient root mean square (RMS). For calculating the count values, a costume mask was applied to the fungal cells on the auto fluorescence channel (excitation at 488 nm; emission at 505–560 nm). Then, according to the intensity values calculated by the instrument, the microorganisms were divided into subpopulations.

### Extraction of *Kluyveromyces marxianus*-secreted molecules

*Kluyveromyces marxianus* culture was centrifuged at 1100×*g* for 10 min, and the supernatant was collected. The supernatant was then moved to a separating funnel and mixed with an equivalent volume of EA for the extraction. The blend was shaken for 10 min, the organic phase was transferred to a new tube, and the extraction was repeated three times. Next, the extracts were evaporated to remove all fluids and finally dried at the lyophilizer to remove water.

### Isolation of tryptophol acetate

Flash chromatography was performed using Merck 40–63 μm silica gel and appropriate solvent based on thin layer chromatography (TLC). TLC (using Silica gel 60 F_254_ plates, Merck) was carried out using Hexane:EA 70:30 (v/v) solvent mixture; fractions displaying suitable Rf of 0.35 (e.g., containing tryptophol acetate) were selected. Preparative HPLC was performed by a Dionex Ultimate 3000 instrument (Thermo Scientific) using a Luna C18 column, 10 μm (250 × 21.20 mm), at a flow rate of 25 mL/min. All runs used linear gradients of 0.1% aqueous TFA (solvent A) vs 90% acetonitrile containing 0.1% TFA (solvent B). The compound was identified by UV detection at a single wavelength (240 nm).

### Liquid chromatography-mass spectrometry (LC-MS)

The kefir biomass crude extract was dissolved in Acetonitrile and injected to LC-MS to identify molecules that originated from the Kluyveromyces marxianus metabolism. The molecular weight was determined by MS using an LTQ XL Orbitrap with a static nanospray in positive ion mode (Waters Acquity QDA with PDA and QDA detectors) analyzed by Xcalibur and Process software (Thermo Scientific). For LC/MS analyses, a Surveyor Plus HPLC System (Thermo Scientific) was used, equipped with a Luna C18, 5 μm (150 × 4.6 mm) column at a flow rate of 0.5 mL/min, using a mobile phase linear gradient of 0.1% aqueous formic acid (solvent A) and acetonitrile containing 0.1% formic acid (solvent B). Additionally, quantitative determination of tryptophol acetate concentration in the kefir biomass crude extract was implemented. To do so, we diluted the Tryptophol acetate at six concentrations: 0.01, 0.02, 0.05, 0.1, 0.25, and 0.50 mM to generate a calibration curve. We produced a plot of tryptophol acetate peak area vs. concentration and the plot showed a linear relationship.

### Gas chromatography-mass spectrometry (GC-MS)

The kefir biomass crude extract was dissolved in Acetonitrile and injected to Thermo Scientific GC-MS with Trace GC ultra and ITQ with RTX-5 0.25 mm × 0.25 mm × 30 m column to identify molecules that originated from the *Kluyveromyces marxianus* metabolism.

### Procedure for synthesis of tryptophol acetate [44]

#### General information

All the reactions were carried out under the air atmosphere in flame-dried glassware. Syringes were used to transfer anhydrous solvents and liquid chemical reagents. Column chromatographical purifications were performed using SiO2 (120-200 mesh ASTM) purchased from Merck. Tryptophol (indole 3-ethanol) was purchased from Alfa Aesar. Triethyl amine, acetyl chloride, acetic anhydride, and P-toluene sulfonic acid were purchased from Sigma-Aldrich. Above chemicals were used without further purification.

To a stirred solution of tryptophol (5 mmol, 1.0 equiv), Tryethylamine (1.4 mL, 10 mmol, 2.0 equiv) in dry DCM (10 mL), acetic anhydride (0.4 mL, 6 mmol, 1.2 equiv) was added in a drop wise at room temperature and further the reaction mixture was stirred at room temperature for 12 h.

After completion, the reaction mixture was quenched by water (5 mL) and the resulting residue was extracted with ethyl acetate (10 mL, 3 times). The combined organic layers were washed with brine (5% sodium chloride (Merck) in water), dried over anhydrous Na2SO4 (Sigma-Aldrich), and concentrated under reduced pressure. Further purification by silica gel column chromatography using hexane/Ethyl acetate (4:1) as the eluent provided the desired product as a colorless solid. The product was confirmed by comparing their ^1^H NMR data with those reported in previous literature (893 mg; purity 99.9%).

### Determination of quorum sensing activity

The effects of the Kefir biomass crude extract molecule on the following bacteria, *A. tumefaciens* A136, *V. cholerae* MM920 and *V. harveyi* MM30 were assessed as described by Brenier et al. [22]. All the strains were cultured as described in the bacterial culturing section. Each culture was diluted to an absorbance density (OD600) of 0.05 by the appropriate fresh LB medium (Lennox). A clear bottom 96-well microliter plate (Thermo Scientific, Rochester, NY, USA) was prepared with wells containing test compound serially diluted into the LB medium starting with the concentration of 200 μM to 12.5 μM. A total of 100 μL of the diluted cultured cells was added to each well. The control sample contained the bacteria and specific autoinducer molecules without containing the tested compound. Luminescence was measured every 20 min for 19 h with continuous shaking at 30 °C, using a Microtiter Plate Reader (Varioskan Flash, Thermo). Two types of experiments were performed: a competition assay in the presence of 400 pM 3-oxo-C8−HSL (*A. tumefaciens* A136), 900 nM CAI-1 (*V. cholerae* MM920), 200 nM (R)-4,5-dihydroxy-2,3-pentanedione (DPD) (*V. harveyi* MM30) and assay in the absence of these AI’s. This allowed measuring agonistic activity of the crude-extracted molecule. Average luminescence values divided by OD600 values were plotted against the added compound concentrations.

### Biofilm modulation activity of kefir biomass crude extract molecules

*P. aeruginosa* PA01, *Salmonella enterica*, and *Staphylococcus aureus* strains were incubated for 24 h at 37 °C. The bacterial suspensions were diluted 1:10 with fresh LB and incubated for 3 h with the kefir biomass crude extract molecules or with DMSO as a control (refreshment samples). In all cultures, DMSO concentration was up to 1%. Two hundred microliters of the broth medium mix with yogurt extract or with DMSO as control was placed in each well of a 96-well plate (Thermo Scientific, Rochester, NY, USA). Two microliters of refreshment samples was added to each suitable well. The plate incubated for 24 h at 37 °C. After the incubation, the samples washed three times with PBS. For visualizing of viable cells, the bacteria were stained using the BacLight® Dead/Live Kit (Invitrogen, Eugene, OR, USA). This resulted in live cells staining green and dead cells stained red. The stained cells were washed twice with PBS. Biofilm images were taken by CLSM (Olympus, Tokyo, Japan). Image processing was done using IMARIS software (Bitplane, Zurich, Switzerland).

### Biofilm modulation activity of synthetic tryptophol acetate

For the static biofilm assay, overnight cultures of *V. cholerae* strain MM920 were diluted 1:10 in fresh LB medium (Lennox) containing a final concentration of 100 μM Tryptophol acetate or DMSO (up to 1%) as a control. We analyzed two types of samples, one in absence of the *V. cholerae* autoinducer (CAI-1) and the other one in the presence of 900 nM CAI-1.

Biofilms were grown under static (non-shaking) conditions at 30 °C in 96-well plates (Thermo Scientific, Rochester, NY, USA). The use of the synthesized molecules did not adversely impact cell growth by visualizing viable cells stained green and dead cells stained red with the BacLight® Dead/Live Kit (Invitrogen, Eugene, OR, USA). Specifically, no increase in dead cells was observed in the presence of the synthesized molecule compared to control biofilms. The stained cells were washed twice with PBS. Biofilm images were taken by CLSM (Plan-Apochromat 20×/0.8 M27, Zeiss LSM880, Germany).

### Real-time quantitative PCR (RT-qPCR) analysis

RNA was extracted from wild-type strain VC1 cultured in LB medium (Lennox) supplemented with or without 100 μM Tryptophol acetate grown to approximately 1.0OD (600 nm) using the RNA Protect Bacteria reagent and the RNeasy® Mini Kit (Qiagen, Valencia, CA, USA) as per the manufacturer’s instructions, including the on-column DNase I digestion described by the manufacturer. Purified RNA was quantified using a Banalyzer (Eppendorf, Hamburg, Germany). cDNA was synthesized from 1 μg of RNA using PrimeScriptTM RT reagent kit (Takara, Ohtsu, Japan). The reaction was incubated at 37 °C for 30 min, and 2 μl of cDNA was subjected to RT-PCR analysis on an AB Step One Plus PCR system (Applied Biosystems, Carlsbad, CA), using qPCRBIO SyGreen Blue mix Hi-ROX (PCR Biosystems, London, UK). RT-PCR was performed in a 96-well plate (Bio-Rad) in triplicate in a 20-μl volume. The *mdh* gene, regulating malate dehydrogenase catalysis, was used as an endogenous loading control for the reactions. The amount of transcript was analyzed with StepOnePlus Software V2.3 (Applied Biosystems Carlsbad, CA, USA). The primers used for RT-qPCR of endogenous reference gene and target genes are listed in Table 1 (Database accession number from complete genome GenBank: AE003852.1 and NZ_CP028828.1).

**Table 1:** Primers used for RT-qPCR of endogenous reference gene and target genes

Gene	Function	Forward sequences (5′–3′)	Reverse sequences (5′–3′)
*mdh*	Regulate malate dehydrogenase	CTGGCGGCATTGGTCAAGCCC	ACCCGGTGTGACAGGCGCAA
*vpsT*	Vibrio polysacchaired transcriptional regulator	CGCAGTATTCAGATGCTGGTG	GACCTCTTTCGCATCAGGACA
*ctxA*	Cholerae toxin subunit A	AGCAGTCAGGTGGTCTTATGC	CCCGTCTGAGTTCCTCTTGC
*aphA*	Virulence gene regulator	ACCGGGTACGATATAACCAAAGAG	GATGGCTGGCTTTCCAGAAG
*toxT*	Transcription activator of virulence genes	TGACGCATACCCATCGACAG	TCACCAGCTAAAAGCCGAGC
*tcpH*	Toxin-co-regulated pilus	TGTTTGGCTTACCCAGACCG	TTCTGAGAGCTAGGATCTGGC
*tcpP*	Toxin-co-regulated pilus	ATTGCATATCAGTCTGGGTTTGC	TCACTTGGTGCTACATTCATGG

### Cholera toxin (CT) detection using GM1-ELISA assay

GM1 (monosialotetrahexosylganglioside) was seeded and immobilized on 96-well white/clear bottom microtiter plate (Greiner) microtiter plates with the following procedure: GM1 stock solution (2 mg/mL in PBS) was diluted with PBS (final conc. 10 μg/mL). Two hundred microliters of the GM1 solution was added to each well and incubated at 37 °C without shaking for 4–16 h. The plates were washed with PBS (× 3). Bovine serum albumin (BSA) was dissolved in PBS (final conc. 4 mg/mL) and 200 μL of the BSA-PBS was added to each well and incubated at 37 °C without shaking for at least 4 h. The plates were washed with PBS (× 3) and were stored in fridge until use. VC1 wild-type strain with the tested compound (Tryptophol acetate in a concentration of 100 μM) and a control with only bacteria were grown and incubated with aeration and shaking overnight in LB medium at 30 °C. The cultures were spanned-down for 5 min at 5000×*g* and the supernatant was taken and diluted 1:2 with a BSA-PBS 4 mg/mL solution. Two hundred microliters of the diluted supernatant was added in six-replicates to each GM1 coated well and the plate was incubated at 37 °C with gentle shaking for at least 30 min. The plates were washed with PBS (× 3), 200 μL of the rabbit antitoxin serum solution (stock solution diluted 1:999 with BSA-PBS) were added to each well and the plate was incubated at 37 °C with gentle shaking for at least 30 min. The plates were washed with PBS (× 3), 200 μL of the IgG solution (goat anti-rabbit immunoglobulin G (IgG) H&L alkaline phosphatase stock solution (1 mg/mL in DDW) was diluted 1:1499 with BSA-PBS) were added to each well and the plate was incubated at 37 °C with gentle shaking for at least 30 min. Luminol working solution was prepared by making two stock solutions. Stock A was prepared by adding 0.1 mL luminol 250 mM in DMSO, 44 μL coumaric acid 90 mM in DMSO, 1 mL tris-HCl 1 M pH = 8.5 and DDW up to a final volume of 10 mL. Stock B was prepared by adding 6.4 μl hydrogen peroxide 30%, 1 ml Tris-HCl 1 M pH = 8.5 and DDW up to a final volume of 10 mL. The plates were washed with PBS (× 3) and quickly equal volumes of stocks A and B were mixed making the luminol working solution. One hundred microliters of the luminol-working solution was added to each well; the plate was shaken for 1.5–2 min and the luminescence was measured using a Microtiter Plate Reader (Varioskan Flash, Thermo).

### Cholerae toxin expression and its cytotoxicity for HeLa cells

#### Western blot analysis

*V. cholerae* (VC1) cells were grown with or without the presence of 100 μM tryptophol acetate at 30 ^o^C for 16 h. Culture supernatants were obtained by centrifugation of these cultures at 1100x*g* for 10 min. The cell pellets lysed using × 5 Sample buffer (125 mM Tris, 0.25% BPB, 10% 2-Mercaptoethanol, 10% SDS, 50% Glycerol; GenScript, Piscataway, USA) for 5 min at 95 °C.

Protein concentrations for the analyzed cells extract samples were determined using Bio-Rad protein assay and then separated by SDS-PAGE and transferred to a polyvinylidene difluoride membrane using a transfer apparatus according to the manufacturer protocols (Bio-Rad). After incubation with 5% skim milk in TBST (10 mM Tris, pH 8.0, 150 mM NaCl, 0.5% Tween 20) for 60 min, the membrane was washed once with TBST and incubated with primary goat anti-cholerae toxin sub unit B (1:500; 227040, Sigma-Aldrich) for 16 h at 4 °C. Membranes were washed three times for 10 min and incubated with a 1:5000 dilution of horseradish peroxidase-conjugated anti-goat secondary antibodies for 1 h at room temperature (A50-101P, Bethyl Laboratories, Montgomery, USA). Beta-actin used as loading control (1:1000, MP Biomedicals, Santa Ana, CA, USA). Blots were washed three times with TBST and developed with the ECL system (Amersham Biosciences) according to the manufacturer’s protocols.

### Cholerae toxin (CT) extraction

*V. cholerae* (VC1) colonies were inoculated in LB broth (Lennox) with and without the presence of 100 μM tryptophol acetate and incubated for 16 h at 30 ^o^C. Culture supernatants were obtained by centrifugation of these cultures at 1100x*g* for 10 min. The cell pellets were washed with PBS and subjected to sonication for 6 min (30 s on and 30 s off) in ice. After sonication, the cell lysates were centrifuged at 1100x*g* for 5 min at 4 ^o^C, filter sterilized using 0.22 μm filter unit (94427, Tracer) and saved in sterile vials.

### Cytotoxicity assay

HeLa cells were grown in 24-well plates (10769-220, VWR International) DMEM supplemented with 10% fetal bovine serum (FBS), 2 mM glutamine (03-020-1; Biological industries), 1× PSN antibiotic mixture (03-031-1; Biological industries) at 37 ^o^C in humidified 5% CO_2_ incubator for 24 h. Subsequently, the medium was replaced with fresh medium combined with 50 μL extracted cholerae toxin (CT) (CT extractions from *V. cholerae* (VC1) were grown in the presence and absence of tryptophol acetate) indicated above and incubated for 16 h. After incubation, the dead cells were stained with Propidium iodide and viable cells were stained green with Syto 9 (BacLight® Dead/Live Kit, Invitrogen, Eugene, OR, USA) and the cytotoxic effects of these bacterial extracts on HeLa cells were examine using an CLSM (Plan-Apochromat 10×/0.8 M27, Zeiss LSM880, Germany). Excitations were at 488 nm and 561 nm; emission 490–588 nm and 604–735 nm, respectively.

### Immunostaining

HeLa cells were grown on sterile coverslips in 24-well plates using DMEM supplemented with 10% fetal bovine serum and 1× PSN antibiotic mixture at 37 ^o^C in humidified 5% CO_2_ incubator for 24 h. Subsequently, the medium was replaced with fresh medium combined with 50 μL cell lysates either with or without presence of 100 μM tryptophol acetate and incubate for 2 h. After 2 h the cells were fixed using 4% formaldehyde solution (1317681, Bio-Lab) for 10 min, and washed with PBS and blocked with 10% FBS (in PBS) for 15 min. All cells were stained with mouse monoclonal anti-cholerae toxin subunit B antibodies (1:500, 200844, Sigma-Aldrich). Cells were then subjected to a secondary antibody staining using goat anti-mouse secondary antibodies Allophycocyanin-conjugated (1159136-068; Jackson ImmunoResearch, West Grove, PA). Finally, cells were mounted with BacLight™ mounting oil (Invitrogen, Eugene, OR, USA). Cells were imaged using CLSM (Plan-Apochromat 20×/0.8 M27, Zeiss LSM880, Germany).

### Statistical analyses

All results are expressed as mean ± SD as indicated. Statistical analyses were done using OriginLab software. Comparisons between three or more groups were performed using one-way ANOVA followed by Tukey’s post hoc test as indicated. A *p* value of ≤ 0.05 was considered to be statistically significant.

## Supplementary Information

**Additional file 1: Table S1.** Identification of microorganisms in kefir based on a BLAST comparison in the One Codex data platform for applied microbial genomics. **Table S2.** Distribution counts of the microorganism population as analyzed by Imagestream® flow cytometry. **Figure S1.** The effect of tryptophol acetate on bacterial growth. a *Vibrio harveyi* b *Agrobacterium tumefaciens* c *Vibrio cholerae*. Growth curves were recorded without and with tryptophol acetate extracted from the kefir mixture. d Vibrio cholerae grown in the presence of synthesized tryptophol acetate (200μM). **Figure S2.** Effect of tryptophol acetate (in concentration of 200μM) on *Pseudomonas aeruginosa* bacterial growth. **Figure S3.** Characterization of tryptophol acetate. (A) GC-MS chromatogram of the molecule showing the retention time spectra and (B) the characteristic m/z spectra showing the fragmentation mass of 144.1 kDa. (C) ^1^H NMR spectrum: δ 2.05 (3H, s), 3.08 (2H, t, J = 5.2 Hz), 4.40 (2H, t, J = 5.2 Hz), 6.93-7.12 (2H, 6.98 (ddd, J = 8.0, 7.8, 1.2 Hz), 7.07 (ddd, J = 8.0, 7.8, 1.6 Hz)), 7.30-7.36 (2H, 7.33 (dddd, J = 8.0, 1.2, 0.5, 0.5 Hz), 7.32 (t, J = 0.5 Hz)), 7.62 (1H, dddd, J = 8.0, 1.6, 0.5, 0.5 Hz). (D) ^13^C NMR spectrum: (100 MHz, CDCl_3_) δ 171.6, 136.4, 127.5, 122.37, 122.1, 119.5, 118.8, 111.8, 111.4, 64.9, 24.9, 21.2. **Figure S4.** Schemes depicting *V. cholerae* quorum sensing regulation of virulence and biofilm formation in high and low cell densities, respectively.

## Data Availability

The main data supporting the findings of this study are available within the article and in its Supplementary Information. All other data supporting the findings of this study are available from the corresponding authors upon request.
