# Supplementary material for: Cross-kingdom inhibition of bacterial virulence and communication by probiotic yeast metabolites
Source: Microbiome. 2021 Mar 24;9:70. doi: 10.1186/s40168-021-01027-8 (PMC7992341; doi:10.1186/s40168-021-01027-8)
Supplement: Supplementary file 2 — Additional file 1: Table S1. Identification of microorganisms in kefir based on a BLAST comparison in the One Codex data platform for applied microbial genomics. Table S2. Distribution counts of the microorganism population as analyzed by Imagestream® flow cytometry. Figure S1. The effect of tryptophol acetate on bacterial growth. a Vibrio harveyi b Agrobacterium tumefaciens c Vibrio cholerae. Growth curves were recorded without and with tryptophol acetate extracted from the kefir mixture. d Vibrio cholerae grown in the presence of synthesized tryptophol acetate (200μM). Figure S2. Effect of tryptophol acetate (in concentration of 200μM) on Pseudomonas aeruginosa bacterial growth. Figure S3. Characterization of tryptophol acetate. (A) GC-MS chromatogram of the molecule showing the retention time spectra and (B) the characteristic m/z spectra showing the fragmentation mass of 144.1 kDa. (C) 1H NMR spectrum: δ 2.05 (3H, s), 3.08 (2H, t, J = 5.2 Hz), 4.40 (2H, t, J = 5.2 Hz), 6.93-7.12 (2H, 6.98 (ddd, J = 8.0, 7.8, 1.2 Hz), 7.07 (ddd, J = 8.0, 7.8, 1.6 Hz)), 7.30-7.36 (2H, 7.33 (dddd, J = 8.0, 1.2, 0.5, 0.5 Hz), 7.32 (t, J = 0.5 Hz)), 7.62 (1H, dddd, J = 8.0, 1.6, 0.5, 0.5 Hz). (D) 13C NMR spectrum: (100 MHz, CDCl3) δ 171.6, 136.4, 127.5, 122.37, 122.1, 119.5, 118.8, 111.8, 111.4, 64.9, 24.9, 21.2. Figure S4. Schemes depicting V. cholerae quorum sensing regulation of virulence and biofilm formation in high and low cell densities, respectively. [file 40168_2021_1027_MOESM2_ESM.docx]

**Supplementary Materials**


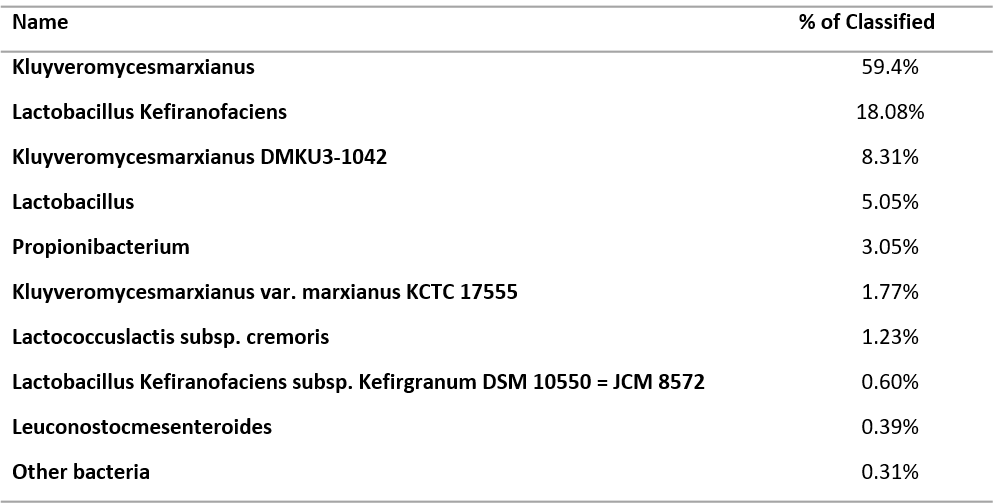


**Table S1.** Identification of microorganisms in kefir based on a BLAST comparison in the One Codex data platform for applied microbial genomics.

|  | Population area in Figure 1B | Count | %Gated |
| --- | --- | --- | --- |
| All |  | 19380 | 100 |
| Bacteria | i | 16832 | 86.85 |
| Fungus | ii | 966 | 4.98 |
| Aggregation of Bacteria & Fungus | iii | 1482 | 7.65 |
|  | iv | 100 | 0.52 |

**Table S2.** Distribution counts of the microorganism population as analyzed by Imagestream® flow cytometry.


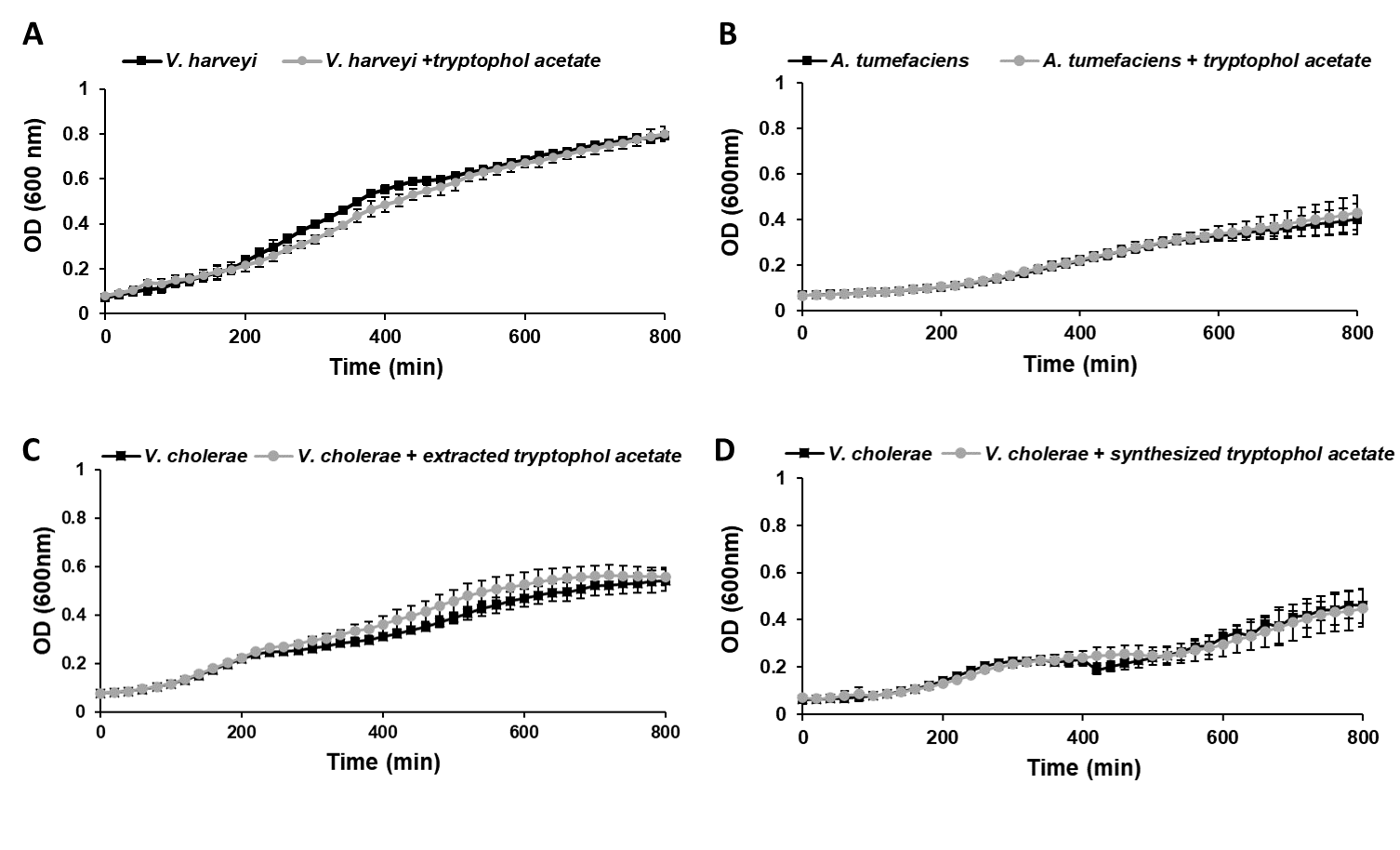


**Figure S1:** **The effect of tryptophol acetate on bacterial growth**. **a** *Vibrio harveyi* **b** *Agrobacterium tumefaciens* **c** *Vibrio cholerae***.** Growth curves were recorded without and with tryptophol acetate extracted from the kefir mixture. **d** Vibrio cholerae grown in the presence of synthesized tryptophol acetate (200µM).


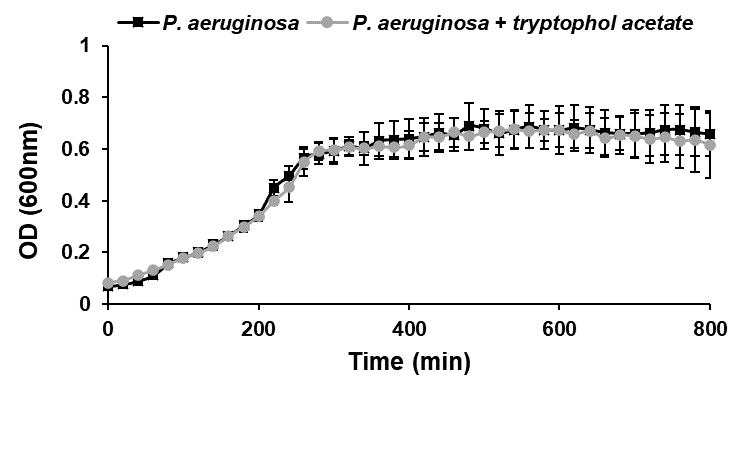


**Figure S2**: effect of tryptophol acetate (in concentration of 200µM) on *Pseudomonas aeruginosa* bacterial growth.


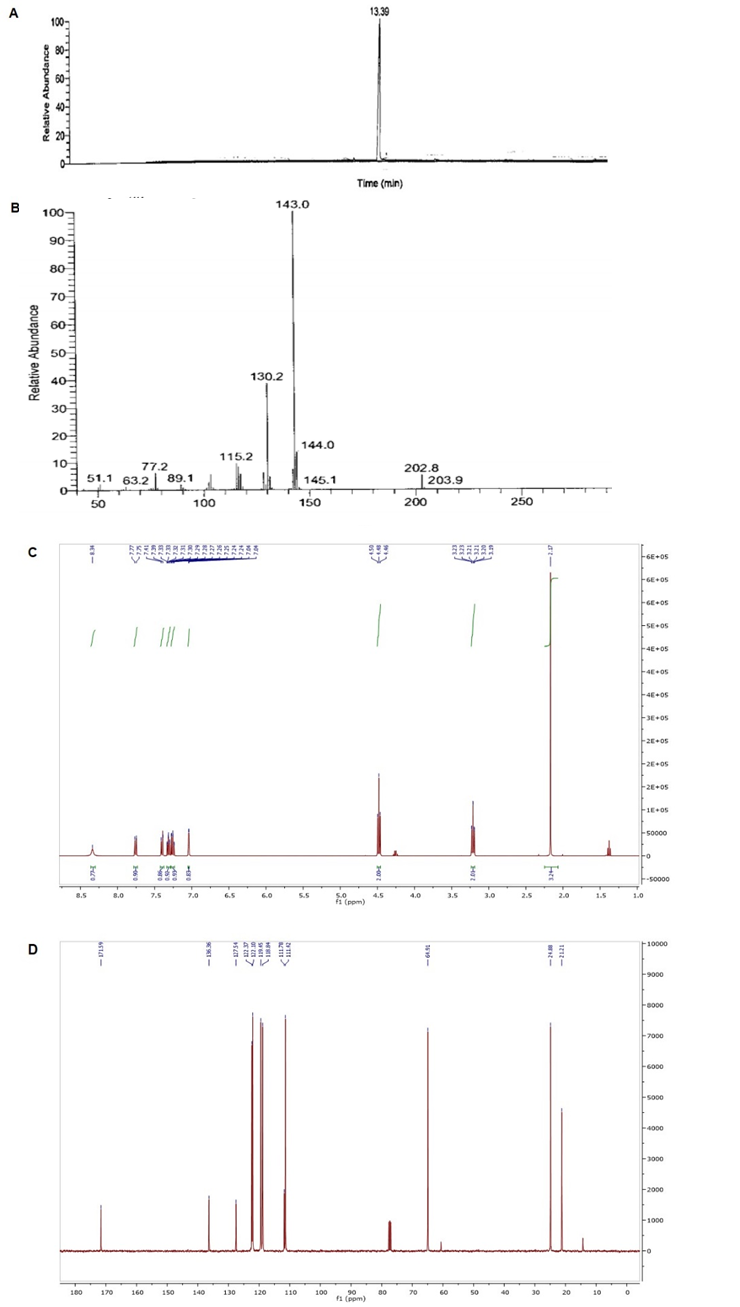


**Figure S3** **Characterization of tryptophol acetate**. (**A**) GC-MS chromatogram of the molecule showing the retention time spectra and (**B**) the characteristic m/z spectra showing the fragmentation mass of 144.1 kDa. (**C**) ^1^H NMR spectrum: δ 2.05 (3H, s), 3.08 (2H, t, J = 5.2 Hz), 4.40 (2H, t, J = 5.2 Hz), 6.93-7.12 (2H, 6.98 (ddd, J = 8.0, 7.8, 1.2 Hz), 7.07 (ddd, J = 8.0, 7.8, 1.6 Hz)), 7.30-7.36 (2H, 7.33 (dddd, J = 8.0, 1.2, 0.5, 0.5 Hz), 7.32 (t, J = 0.5 Hz)), 7.62 (1H, dddd, J = 8.0, 1.6, 0.5, 0.5 Hz). (**D**) ^13^C NMR spectrum: (100 MHz, CDCl_3_) δ 171.6, 136.4, 127.5, 122.37, 122.1, 119.5, 118.8, 111.8, 111.4, 64.9, 24.9, 21.2.

**
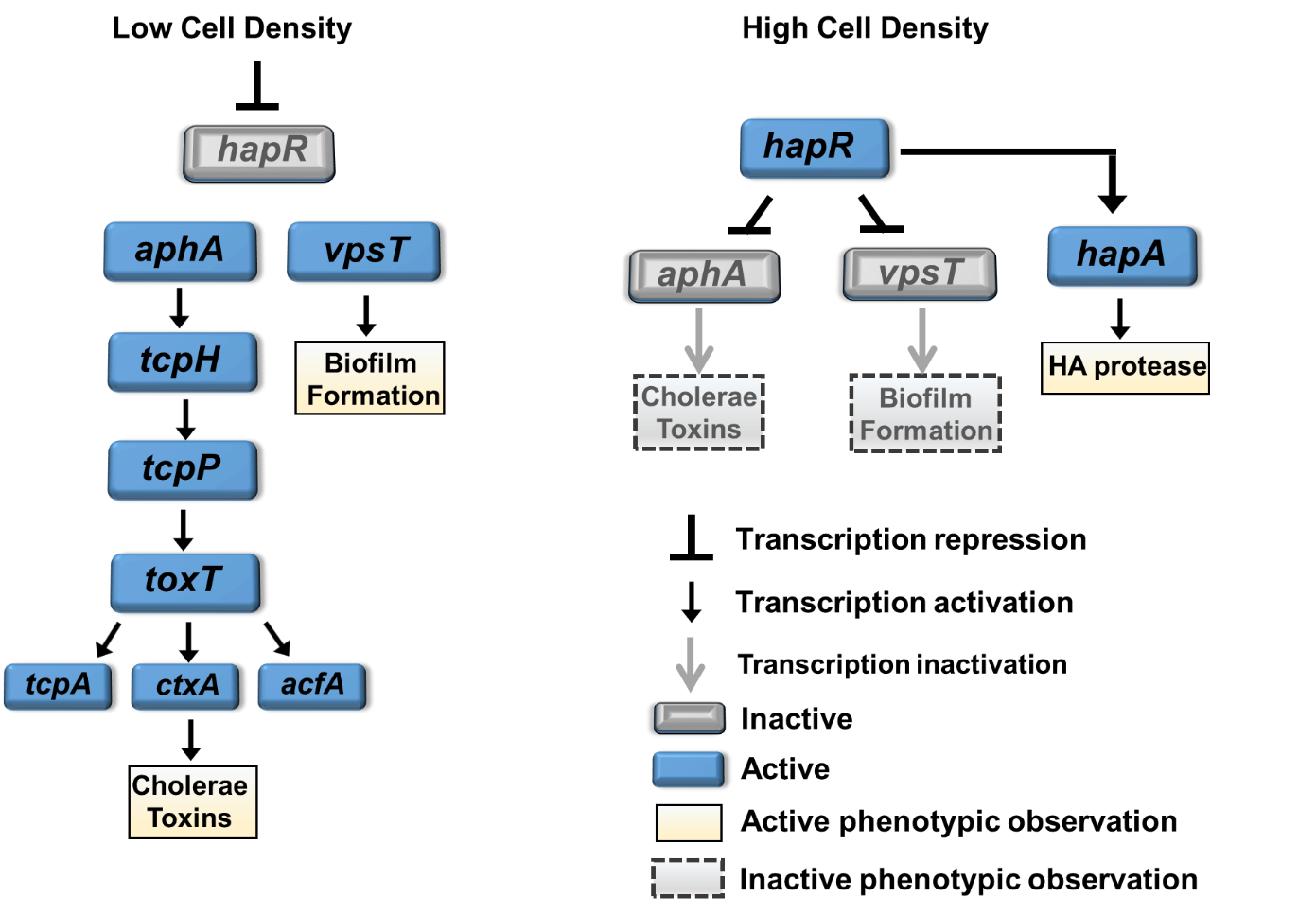
**

**Figure S4:** Schemes depicting *V. cholerae* quorum sensing regulation of virulence and biofilm formation in high and low cell densities, respectively.
